# GM1 ganglioside prevents axonal regeneration inhibition and cognitive deficits in a mouse model of traumatic brain injury

**DOI:** 10.1038/s41598-018-31623-y

**Published:** 2018-09-06

**Authors:** Amit Benady, Dor Freidin, Chaim G. Pick, Vardit Rubovitch

**Affiliations:** 1 Department of Anatomy and Anthropology, Sackler Faculty of Medicine, Tel-Aviv University, Tel-Aviv, 69978 Israel; 2 Sagol School of Neuroscience, Tel Aviv University, Tel Aviv, Israel

## Abstract

Traumatic Brain Injury (TBI) is one of the most common causes of neurological damage in young populations. It has been previously suggested that one of the mechanisms that underlie brain injury is Axonal Outgrowth Inhibition (AOI) that is caused by altered composition of the gangliosides on the axon surface. In the present study, we have found a significant reduction of GM1 ganglioside levels in the cortex in a closed head traumatic brain injury model of a mouse, induced by a weight drop device. In addition, axonal regeneration in the brains of the injured mice was affected as seen by the expression of the axonal marker pNF-H and the growth cones (visualized by F-actin and β-III-tubulin). NeuN immunostaining revealed mTBI-induced damage to neuronal survival. Finally, as expected, spatial and visual memories (measured by the Y-maze and the Novel Object Recognition tests, respectively) were also damaged 7 and 30 days post injury. A single low dose of GM1 shortly after the injury (2 mg/kg; IP) prevented all of the deficits mentioned above. These results reveal additional insights into the neuroprotective characteristics of GM1 in prevention of biochemical, cellular and cognitive changes caused by trauma, and may suggest a potential intervention for mTBI.

## Introduction

Traumatic Brain Injury (TBI) occurs when an object or external force hits the head. The main causes of injury are road accidents, falls, assaults, and sport injuries^1^. Amongst the population under the age of 50, TBI is the most common neurological disorder^2–4^. The effects of TBI can be temporary or permanent and can cause physiological, cognitive, motor and behavioral damage, which range on the spectrum between total lack of symptoms to severe deficits and death^5^. Despite the scope of the phenomenon, there is still no complete understanding of the processes that occur in the brain after the injury at the molecular and biochemical level. Mild Traumatic Brain Injury (mTBI), which accounts for over 80% of head injuries is difficult to diagnose because routine tests, including imaging, fail to demonstrate changes in brain structure^6,7^.

The pathophysiology of traumatic brain injury can be divided into primary and secondary injury mechanisms. The initial injury is due directly to the physical injury and may result in intracranial or extracranial hemorrhage following damage to the blood vessels^8^. In addition, damage to the brain tissue and the brain blood barrier (BBB) may occur^9,10^. As a result, cells may die an apoptotic or necrotic death immediately after injury. Following these events, the secondary injury occurs within a period of hours to weeks after the first injury^8^, which will then initiate a series of inflammatory reactions. Mostly, the secondary injury will be more severe and complex than the primary, and will include anatomical, cellular, molecular and behavioral changes. When the neuronal cells fail to overcome these damages, secondary damage will cause cell death^11^. Since it takes time for secondary damage to occur, a “window of opportunity” is created in which a substance or medication that can act on one or more pathological pathways that underlie the damage, may be administered.

In contrast to the peripheral nervous system, the central nervous system is usually not accompanied by significant regeneration due to the inhibitory effect of extracellular factors that accumulate in the injury site, including myelin proteins, astrocytes, oligodendrocytes and microglia. Experiments have shown that in some artificial environments the axons can regenerate, so it can be concluded that the main problem with the regeneration of axons in the central nervous system is the inhibitory environment and the scar tissue created after the injury^12^. One of the suggested explanations is the axonal outgrowth inhibition (AOI), which occurs partly because of a change in the composition of the gangliosides on the axon surface and partly by the accumulation of axonal regeneration inhibitors (ARIs) at the site of the damage. This accumulation of proteins mainly involves Myelin-Associated-Glycoprotein (MAG), NOGO and Oligodendrocyte-Myelin glycoprotein (OMgp). These proteins may bind to the gangliosides, which act as receptors on the axon, and signal them to delay the regeneration^13–15^.

Ganglioside is a fatty molecule that is very common in the brain and is located on the plasma membrane. It is made up of glycosphingolipid (ceramide and oligosaccharide) and is connected to a chain of sugars. There are more than 60 different types of glycosphingolipids that differ mainly in the quantity and location of NANA residues. About 96% of brain gangliosides are divided into four types: GM1, GD1a, GD1b, GT1b, where each of them has different affinity for myelin proteins. Changes in gangliosides expression are expressed in neuronal disorders and degeneration of axons^16^. The first evidence of ganglioside involvement in inhibition of axon regeneration was primarily from *in-vitro* experiments on isolated neurons from rats and mice^17^. Increasing the knowledge about the molecular connections of gangliosides can improve understanding of axon-myelin stability and provide an opportunity to increase the recovery after traumatic injury to the nervous system. In studies performed in our laboratory, it was shown that 72 hours after a blast injury in mice, GM1 ganglioside levels were significantly lower than the levels in the control group. The administration of GM1 ganglioside immediately after the explosion (2 mg/kg, IP) not only prevented this decrease but also caused increased expression of the ganglioside and its arrangement in cortices. In addition, it was shown that neither the impact of the blast nor the administration of GM1 ganglioside affected the GD1a ganglioside levels^18^.

The main goal of this study was to demonstrate the reduction of GM1 after a closed head weight drop model for mTBI and the possible neuroprotective effect of a single low dose of GM1 administration to prevent the cognitive deficits and the biochemical changes.

## Experimental Procedures

**Figure 1:**
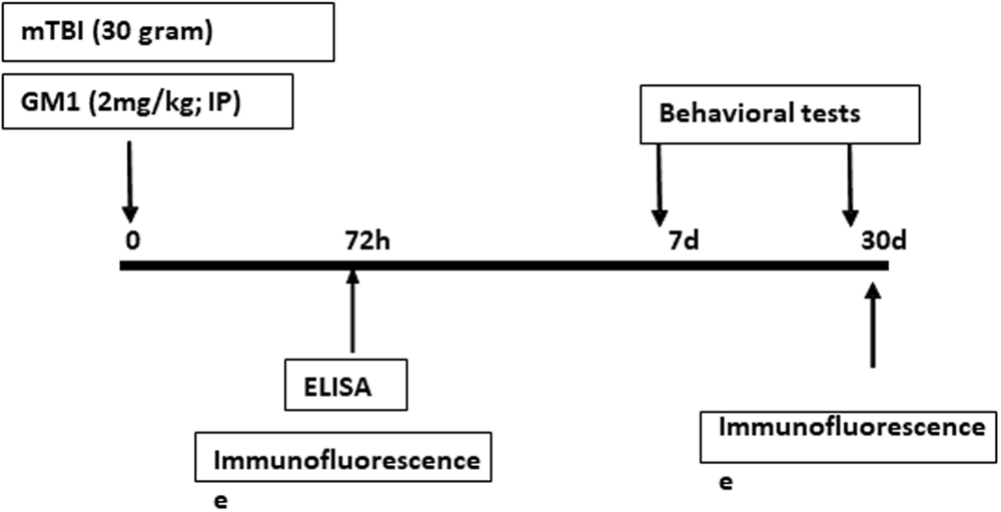
Timeline of the experimental procedures following mTBI. Six week old ICR mice were subjected to 30 gr weight drop impact as described in the ‘experimental procedures’. GM1 (2 mg/kg) was injected post injury. We performed the behavioral tests 1-week or 30 days post injury while for biochemistry, immunohistochemistry and ELISA mice were sacrificed 72 hrs or 30 days (NeuN) post injury as described.

^*^Figure 1 shows the time line of the experimental procedures.

### Animal studies

ICR male mice, ages 6–8 weeks, weighing 30–40 grams, were purchased from Envigo RMS Israel. The Sackler Commission on Animal Experimentation approved the trial protocol 01-10-034 according to the Guidelines for Animal Experimentation of the National Institutes of Health (DHEW publication 23–85, revised, 1995). The mice were kept at room temperature at a light/dark cycle of 12 hours, 5 mice in a standard plastic cage (32 × 21.5 × 12 cm^3^). Purina rodent chow and water were freely accessible (ad libitum). The cages bedding was sawdust which was replaced twice a week, in parallel for all the cages. After the animals arrived at the laboratory, they were given three days of recovery from the transport and for acclimatization in the new location. Two days before the experiment all the cages were transferred to the experimental room for the purpose of getting used to the new environment, and reducing anxiety.

### Induction of head injury in mice

The head injury in this experiment was performed according to the closed head weight drop model. In this model a fixed weight is released for a free fall according to a defined path. The weight and the height from which it is imposed determines the severity of the injury, and it can range from a mild level of injury to severe brain injury. In the pre-induction stage, the mice were anesthetized by inhaling Isophlorane vapor for several minutes. At the time of induction, the mice were kept in such a way as to direct the injury from the frontal lateral direction on the right side of the mouse’s head, at an equal distance between the eye and the right ear. The mice were placed on a spongy surface that allows the movement of the head parallel to the injury plane at the time of the weight fall and thus mimics a head injury that occurs during a car accident. The head injury was induced by using the concussive head trauma device, which was been described previously in our lab^4,19^. The device is a hollow metal tube (with an internal diameter of 13 mm) that is placed vertically above the head of the mouse. A metal weight (30 g) is inserted into the metal pipe, which falls in a free fall of 80 cm. This model was chosen because it best describes traumatic head injuries such as road accidents or falls, as it imposes a diffuse and non-specific injury, as traumatic head injuries are often characterized.

### Administration of GM1

The mice in the mTBI + GM1 and GM1 groups received one injection of 0.1 ml per 10 g mouse weight (ganglioside GM1, Calbiochem USA) shortly after the injury. The substance was dissolved in dimethysulfoxide (DMSO) and then diluted with saline to final concentration of 2 mg/kg and injected IP (Intra Peritoneum). Mice in the other two groups received a vehicle injection (DMSO) of the same volume.

#### Biochemistry

Immunohistochemistry: 72 h post mTBI, mice (n = 3–4 for each group) were anesthetized with a combination of ketamine (90 mg/kg) and xylazine (10 mg/kg) and perfused transcardially with PBS and then with 4% paraformaldehyde (PFA) in 0.1 M phosphate buffer, pH 7.4. Their brains were removed, fixed overnight in 4% PFA in 0.1 M phosphate buffer, pH 7.4, and then placed in 30% sucrose for 48 h. Frozen coronal sections (30μm) were then cut on a sliding microtome and collected serially. The free-floating sections were first blocked by incubation with 0.1% Triton X-100 in phosphate-buffered saline and 10% normal horse serum for 1 h at 25 °C. Primary antibodies (phosphorylated NF-H, SM1-31R Covance; USA, Diluted 1:1000 in incubation buffer; rabbit anti GD1a ganglioside ab23943; abcam, Diluted 1:1000 in incubation buffer; rabbit anti F-actin ab205; mouse anti beta III tubulin Abcam, Cambridge, UK) diluted in PBST and 2% normal horse serum, and incubated with the sections for 48 h at 4 °C. Control slices were stained by omitting primary antibodies. After being rinsed in PBST the sections were incubated for 1 h at 25 °C with Donkey anti mouse IgG H & L (Alexa Fluor®488) and Goat Anti-Rabbit IgG H & L (Alexa Fluor® 594)After rinses in PBST, free-floating sections were mounted on dry gelatin-coated slides, and fluorescence was visualized using a Leica SP8 confocal microscope (Leica, Germany). Excitation light was provided by the 488 nm line of argon lasers for the Alexa-488 fluorophore, the 543 nm line of HeNe lasers for the Alexa-594 fluorophore and 405 excitations for DAPI. All the fluorescence images for a certain figure were taken with the same scanning parameters. The quantification of pNF-H, NeuN, F-actin and beta III tubulin expression were done using the Imaris software, (Bitplane AG, Zurich, Switzerland). Overall, we had 3–4 sections (different bregmas) from each brain. Images were taken from 3 fields (from each section) at the post central cortex and the hippocampus. The images from each section and from each animal were averaged for the final value (signal intensity for pNF-H, F-Actin and βIII-Tubulin; number of stained cells/500 μm^2^ for NeuN).

#### Indirect enzyme-linked immunoassay (ELISA)

ELISA was used for GM1ganglioside quantification and was performed according to ABCAM protocol (UK; http://www.abcam.com/ps/pdf/protocols/Indirect%20ELISA%20protocol.pdf). 96 Well plates (Corning, Sigma-Aldrich) were coated with the cortex samples and remained overnight at 4 °C. After washing 3 times with PBS, 200 μL blocking buffer (5% non-fat dry milk in PBS) was added and incubated for 2 h in R.T. Rabbit anti ganglioside GM1 (ab23943 Abcam, Cambridge, UK) diluted 1:1000 (Dilutions were made in PBST and 2% normal horse serum) was applied on the wells for 2 h in R.T. Following washing (5 times) peroxidase-conjugated Goat Anti-Rabbit secondary antibody (Jackson Immuno-Research PA/USA) was added for 90 min. HRP chromogen (TMB, R & D systems) was then added after washing. The results were calculated using a four-parameter curve fit and expressed as ng/ml, then normalized relative to the control (100%).

#### Cognitive assessments

Y-maze: This test examines short-term spatial memory and relies on the animal’s preference for exploring a new place. The maze is made of a black plexiglass with three arms (8 × 15 × 30 cm), including a 120 ° angle. One arm was randomly chosen to be the “start” arm. The test is made up of two stages^9,20^. In the first stage, each animal is placed on the outer edge of the arm called the “start” arm. One of the two remaining arms is blocked. It is called a “new” arm while the other arm is called an “old” arm. The animal is given 5 minutes to wander around freely in both open arms. When time finishes, it was returned to its cage for 2 minutes in which the maze was cleaned with ethanol to remove odors left behind by the animal. In the second stage, after 2 minutes the animal was returned to the maze, when this time it was free to wander around all three arms for 2 minutes. During this period, the animal’s time was measured by each arm to see if the animal had differentiated between a “new” arm and an “old” arm. Between each animal we replaced the “new” arm in order to reduce the bias of the results because of preference for a particular arm, if any. In this test naive animals are expected to show, in the second stage of the experiment, a preference to stay in the “new” arm compared to staying in the “old” arm, thinking that the animals remember the “old” arm and because of their curiosity are interested in being in a new place.

The Aggleton index^21^ is calculated to estimate the spatial memory of animals according to the following formula:

$${\rm{Preference}}\,{\rm{index}}=\frac{(\mathrm{time}\,{\rm{in}}\,\mbox{''}{\rm{new}}\mbox{''}\,\mathrm{arm}\,-\,\mathrm{time}\,{\rm{in}}\,\mbox{''}{\rm{old}}\mbox{''}\,\mathrm{arm})}{(\mathrm{time}\,{\rm{in}}\,\mbox{''}{\rm{new}}\mbox{''}\,{\rm{arm}}+{\rm{time}}\,{\rm{in}}\,\mbox{''}{\rm{old}}\mbox{''}\,\mathrm{arm})}$$

An animal with good spatial memory would have a high Preference index, while an animal with a damaged spatial memory would have a low Preference index. Animals who were present at the old and new arms less than 10% of the total time of the test (i.e. less than 12 seconds in both arms) were excluded from statistical calculations, since when a mouse does not access the arms at all, it is not possible to estimate its spatial memory level.

Novel Object Recognition (NOR): This test examines the visual memory of the animal based on the natural curiosity that exists in rodents for new objects. The maze is built in the shape of a square surface (60 cm × 60 cm) and has high walls (20 cm) The Novel Object Recognition test consists of three stages^22^:

1. Adjustment stage - The mouse is inserted into an empty surface for 5 minutes to learn the surface itself.
2. Learning stage (24 hours after acclimatization) - The mouse is inserted into a surface where two identical objects (A) are placed for 5 minutes to recognize them. We will define them as “old” objects.
3. The test stage (24 hours after learning stage) - The mouse is inserted into the surface where two objects are placed, one is known from the learning phase (A) and one is “new” (B), for 5 minutes.

Between one animal and the other, the surface and the objects were cleaned with ethanol to mask odors that the animals left behind.

The Aggelton index^21^ is calculated to assess the extent of learning and visual memory of animals according to the following formula:

$${\rm{Preference}}\,{\rm{index}}=\frac{(\mathrm{time}\,{\rm{near}}\,\mbox{''}{\rm{new}}\mbox{''}\,\mathrm{object}\,-\,\mathrm{time}\,{\rm{near}}\,\mbox{''}{\rm{old}}\mbox{''}\,\mathrm{object})}{(\mathrm{time}\,{\rm{near}}\,\mbox{''}{\rm{new}}\mbox{''}\,{\rm{object}}+{\rm{time}}\,{\rm{near}}\,\mbox{''}{\rm{old}}\mbox{''}\,\mathrm{object})}$$

A higher preference index indicates better learning. Animals who were present at the objects less than 10% of the total time of the test (i.e. less than 30 seconds next to the two objects together) were excluded from statistical calculations, since when a mouse does not access objects at all, it is not possible to estimate its visual memory level.

### Data analysis

All values are presented as mean values ± standard error to average. Significance was calculated in the ANOVA (analysis of variance) tests for continuous variables. To compare more detailed data, we used Fisher LSD post hoc tests. All the tests were performed in SPSS_20. Statistically significant differences between the averages will be marked by asterisk *P ≤ 0.05, **P ≤ 0.01, ***P ≤ 0.001.

## Results

### mTBI significantly reduced the expression of GM1 ganglioside in the brains of the injured mice

**Figure 2:**
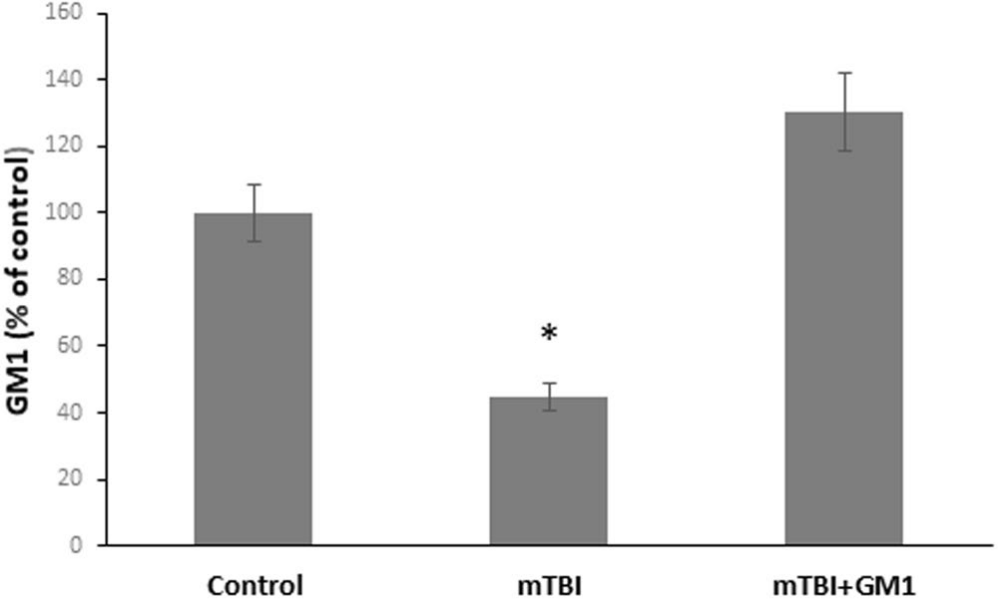
mTBI significantly reduced the expression of GM1 in the cortex. Indirect enzyme-linked immunoassay (ELISA) test also revealed a significant reduction in GM1 content in the cortex of the injured mice compared with control (72 hrs post injury), which was significantly prevented by the administration of GM1. [F (2,15) = 5.153; ^*^p < 0.05]. The results were calculated using a four-parameter curve fit and expressed as ng/ml, then normalized relative to the control (100%).

Our basic hypothesis refers to the alteration in ganglioside expression as one of the mechanisms that underlie the damage in the brains of mTBI mice. Hence, we evaluated the expression of GM1 using an Indirect enzyme-linked immunoassay (ELISA) test that revealed a significant reduction in GM1 content in the cortex of the injured mice 72 hrs post mTBI compared with control mice (21.08 ± 4.23 and 47.21 ± 8.7, respectively). GM1 administration shortly after the injury prevented this decline (61.6 ± 11.6). Fisher LSD post-hoc analysis revealed **p < 0.001. Statistical significance was analyzed by one-way ANOVA test [F (2,15) = 5.153; *p < 0.05]. (Fig. 2).

### mTBI induced a significant decline in the axonal marker pNF-H in the cortex and in the hippocampus

**Figure 3:**
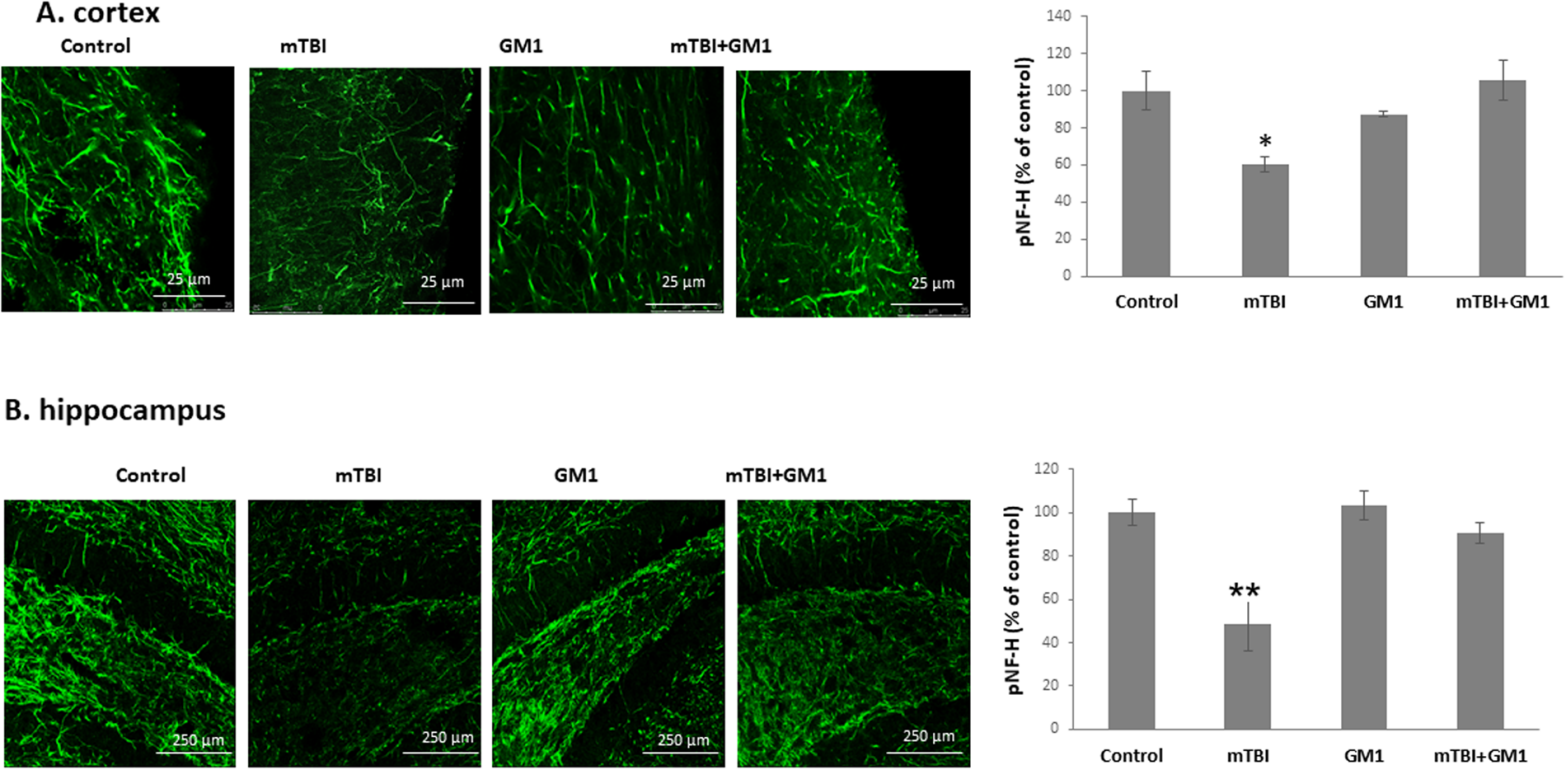
The neuroprotective effect of GM1 administration on axonal regeneration as seen by phosphorylated NF-H Phosphorylated NF-H (green) positive neurons in the cortex (**A**) and hippocampus (**B**). [F(3,10) = 6.109; ^*^p < 0.05]; [F(3,8) = 10.391; ^**^p < 0.01]. Values are mean ± SEM, of n = 4–6 mouse brains. The results are the average of the signal intensity.

Since we suggest AOI (axonal outgrowth inhibition) as the result of GM1 decline, the next experiments were aimed at evaluating the effect of GM1 administration on the expression of the phosphorylated NF-H (the axonal heavy neurofilaments) as seen in immunofluorescence microscopy. As expected in our model, mTBI significantly reduced the expression of pNF-H in the cortex 72 hrs post injury. pNF-H expression declined from 1152 ± 117.1 in control brains (n = 5) to 692 ± 47.9 in mTBI brains (n = 4). GM1 prevented this decline (1218 ± 12.5; n = 6). Fisher LSD post-hoc analysis revealed **p < 0.001. Statistical significance was tested by one-way ANOVA [F(3,10) = 6.109; *p < 0.05] followed by the LSD post hoc analysis (Fig. 3A).

Similarly, GM1 blocked the reduction of pNF-H in the hippocampi of mTBI injured mice 72 hrs post injury. The signal intensity declined from 7710.9 ± 461.7 in control brains (n = 5) to 3729 ± 928.1 in mTBI mice (n = 4). Fisher LSD post-hoc analysis revealed **p < 0.001. GM1 injection prevented this decline: (6992.7 ± 350.3; n = 6). Fisher LSD post-hoc analysis revealed **p < 0.001. Statistical significance was tested by one-way ANOVA test [F(3,8) = 10.391; **p < 0.01] followed by Fisher LSD post hoc (Fig. 3B).

### The effect of GM1 on the axonal regeneration be viewing growth cones in the cortex of mTBI mice

**Figure 4:**
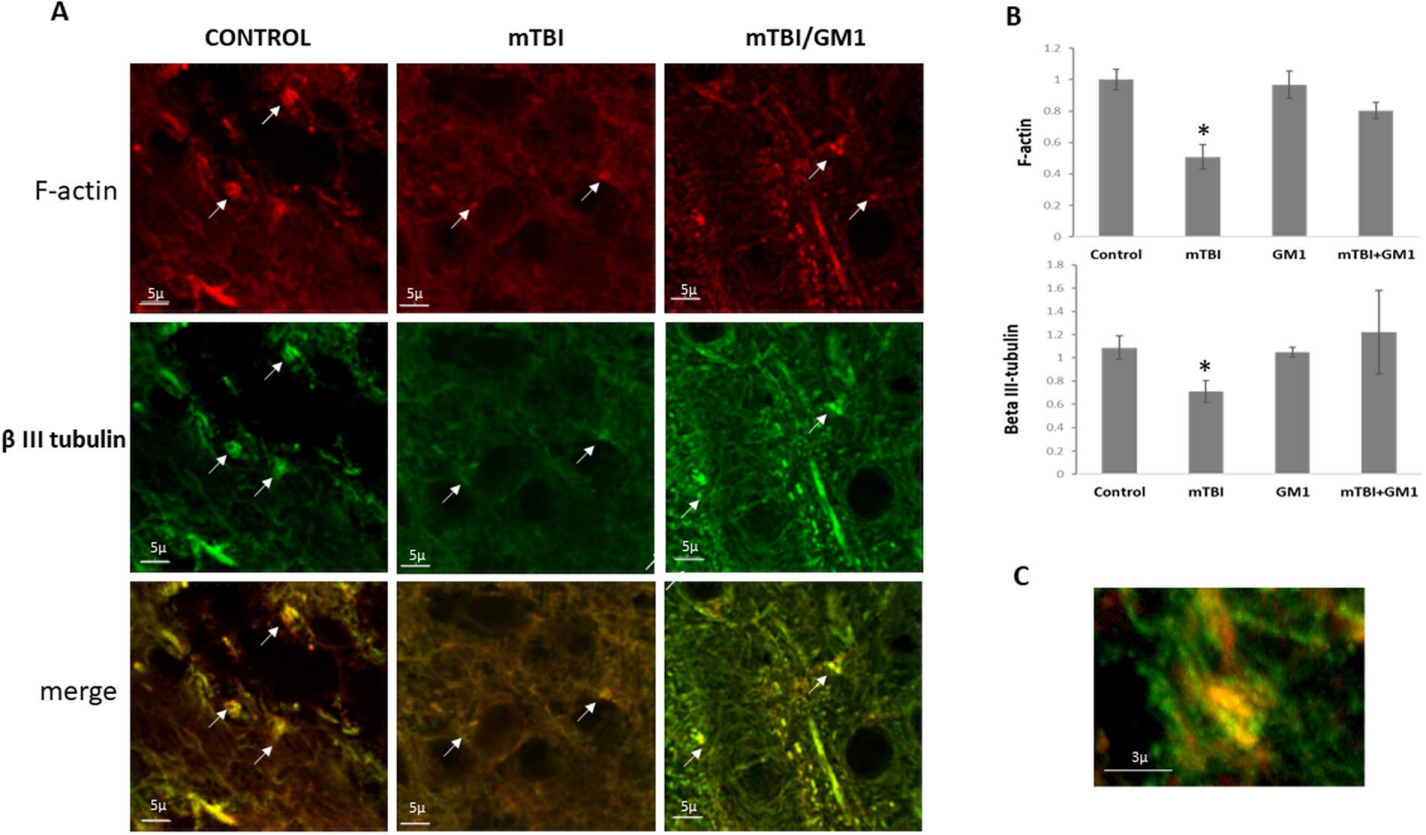
The neuroprotective effect of GM1 administration on growth cones damage Brain sliced were stained for F-actin and β-tubulin to identify cortical growth cones. Representative images of cortex slices immuno-stained with F-actin and βIII-tubulin antibodies which identify growth cones. TBI induced a significant shrinking/collapse of the growth cones (size and expression). GM1 treatment prevented this effect and these structures were preserved. (the white arrows point to growth cones). [F(3,8) = 9.98; ^*^p < 0.05]; [F(2,13) = 6.6; ^*^p < 0.05]; n = 3. The results are the average of the signal intensity.

In order to evaluate the axonal regeneration we double stained the brain slices with antibodies raised against F-actin and β-tubulin III. We evaluated the expression of these proteins by immunofluorescence analysis. F-actin stains the growth cones and serves as a marker for axonal regeneration in the mouse brain together with β-tubulin III. Figure 4 shows that mTBI induced a significant decline in F-actin expression 72 hrs post injury (0.5 ± 0.07) compared to the control group (1 ± 0.06); Fisher LSD post-hoc analysis revealed **p < 0.001. This reduction was prevented under the administration of GM1 (0.8 ± 0.05); Fisher LSD post-hoc analysis revealed *p < 0.05. Statistical significance was tested by one-way ANOVA [F(3,8) = 9.98; *p < 0.05] followed by Fisher LSD post hoc (Fig. 4). Similarly, the expression of β-tubulin III was also significantly reduced 72 hrs after mTBI (0.7 ± 0.09) compared to the control group (1.08 ± 0.1). Fisher LSD post-hoc analysis revealed **p < 0.001. This reduction was prevented under the administration of GM1 (1.22 ± 0.35); Fisher LSD post-hoc analysis revealed *p < 0.05. Statistical significance was tested by one-way ANOVA test [F(3,13) = 6.6; *p < 0.05] followed by Fisher LSD post hoc (Fig. 4).

### The Effect of GM1 administration on neuronal survival in the cortex of mTBI group mice

**Figure 5:**
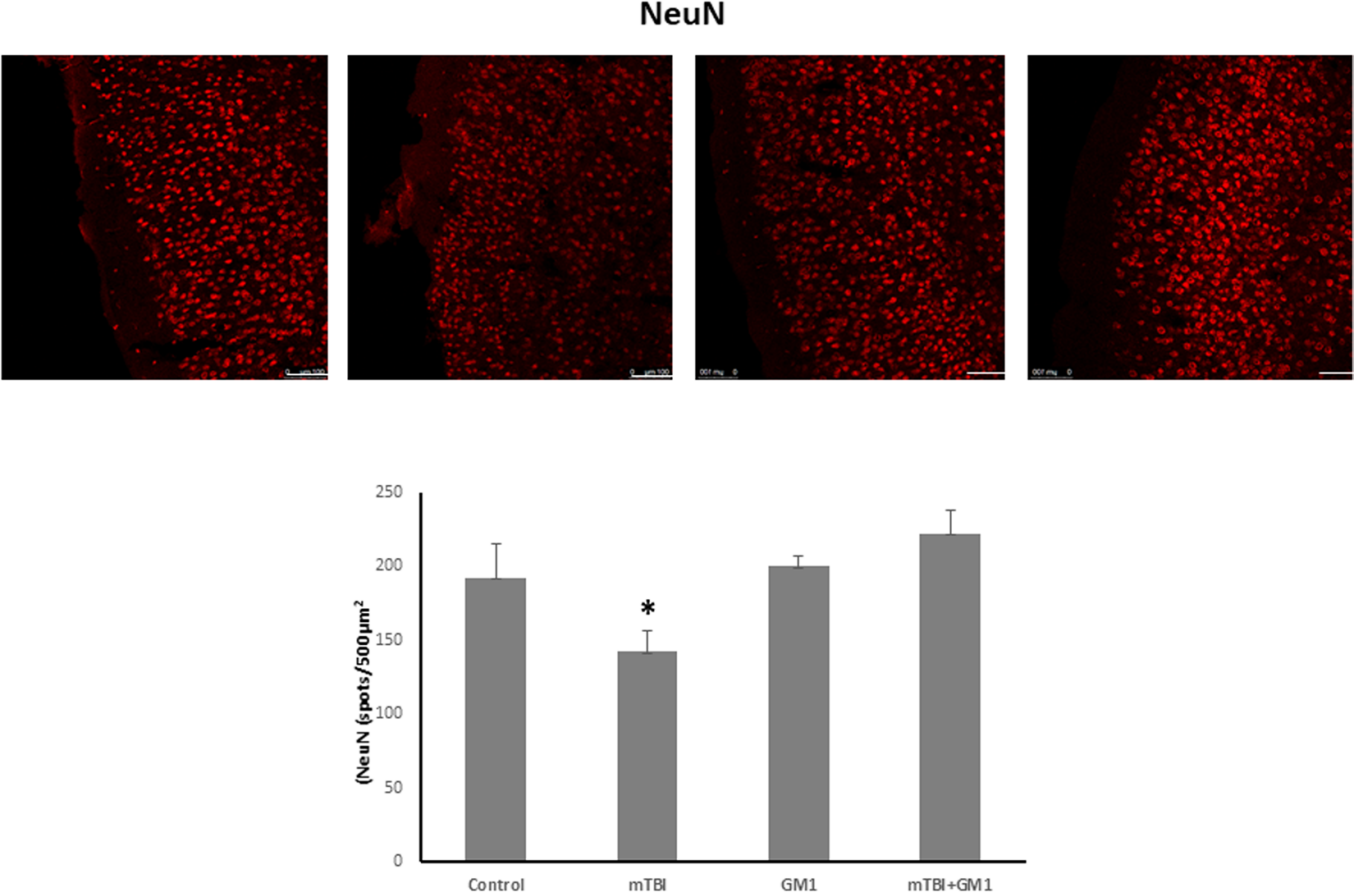
The neuroprotective effect of GM1 administration on neuronal survival. TBI induced a significant reduction on neuronal survival in the cortex 30 days post injury, as seen by NeuN staining. GM1 administration shortly after injury prevented this degeneration. [F(3,10) = 5.558; ^*^p < 0.05]. n = 4–6. The results are the average of the number stained cells/500 μm^2^.

The next experiment was aimed at evaluating the neuronal survival as seen by immunostaining brain slices with antibodies against NeuN (a marker of mature neuronal DNA), 30 days post injury. We also examined whether cell death was prevented by administering GM1 immediately after mTBI. As expected, the results, which are presented in Fig. 5, show a decrease in the number of neurons after mTBI (80.4 ± 12.9) compared to the control group (128.3 ± 9.2). Fisher LSD post-hoc analysis revealed **p < 0.001. This decrease was prevented after the administration of GM1 (134.8 ± 8.3). Fisher LSD post-hoc analysis revealed **p < 0.001. Statistical significance was tested by one-way ANOVA test [F(3,10) = 5.558; *p < 0.05] followed by Fisher LSD post hoc. Thus, we can conclude that GM1 prevented neuronal death in the cortex after mTBI in mice (Fig. 5).

### The effect of GM1 administration on mTBI-induced cognitive deficits in mice

We and others have previously reported that TBI caused a significant damage to cognitive performance. Since our goal was to confirm that this reduction is mediated by gangliosides alternation we examined how the administration of GM1 in a single low dose (2 mg/kg; IP) immediately after the injury affected the cognitive functions both one week and one month after the mTBI. These functions were examined using two behavioral tests: Novel Object Recognition, which examines visual memory, and Y-maze, which examines spatial memory.

#### Novel object recognition test

**Figure 6:**
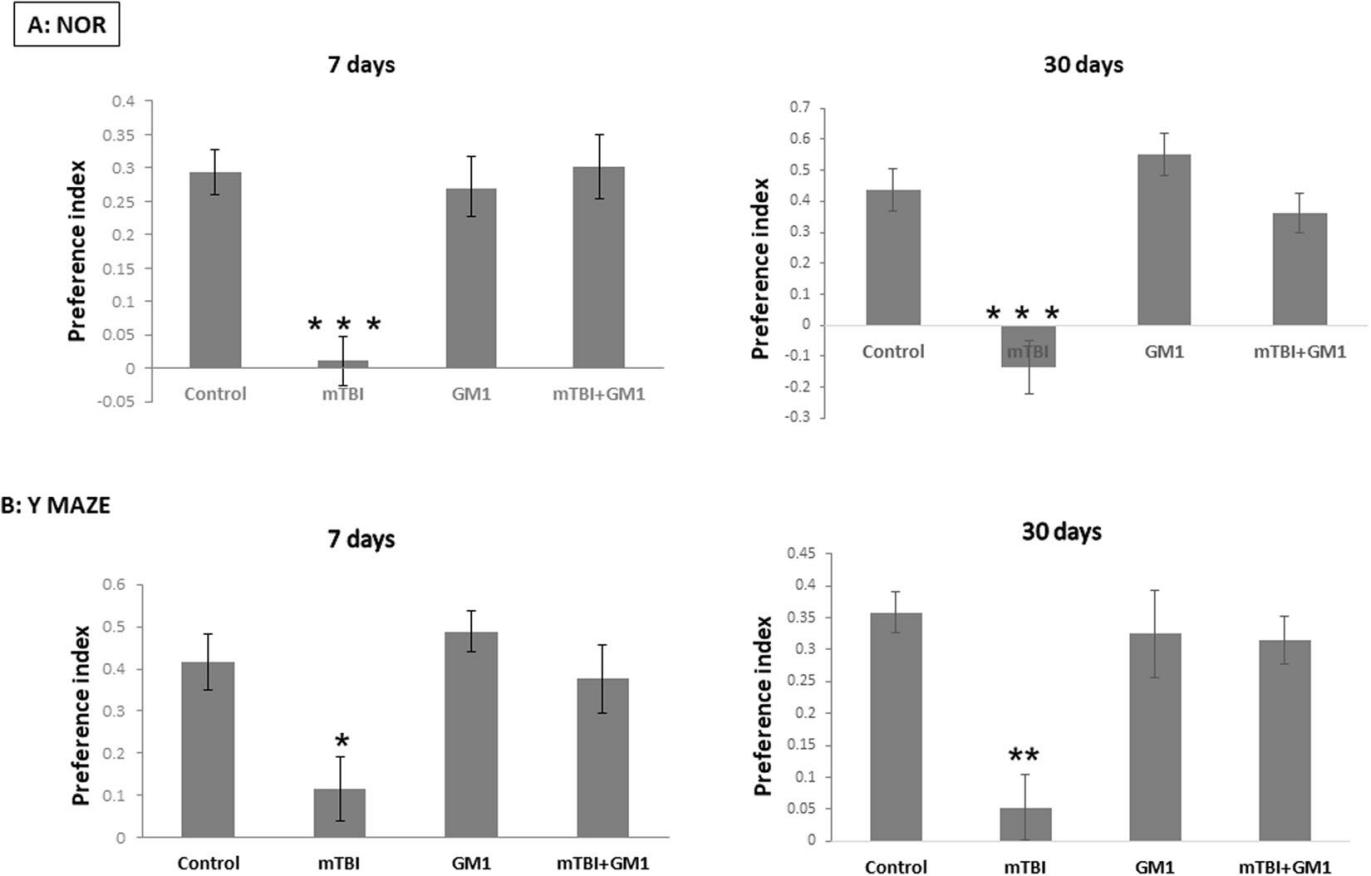
The neuroprotective effect of GM1 administration on the cognitive performance after mTBI The behavioral tests were performed 7 or 30 days post mTBI (separate groups). (**A**) The recognition memory was assessed by the Novel Object Recognition test (NOR) by calculating the relative time that the mice spent near a novel object compared to an old, familiar one (“preference index”, see methods). The significant decrease of preference index in mTBI mice was significantly prevented by the administration of GM1 (2 mg/kg;IP). One way ANOVA revealed significant effect of group: [F(3,34) = 10.361; ^***^p < 0.001] [F (3,27) = 17.476; ^***^p < 0.001]. (**B**) The spatial memory was assessed by the Y-maze test (Y-MAZE) by calculating the relative time that the mice spent in a newly open arm compared to an old, familiar one (“preference index”, see methods). [F (3,43) = 4.897; *p < 0.05]; [F(3,33) = 7.670; ^**^p < 0.01].

The results of this test shown in Fig. 6 present a significant reduction in visual memory in mTBI mice one week after injury (0.01 ± 0.03) compared to the control group (0.29 ± 0.03). Fisher LSD post-hoc analysis revealed ***p < 0.001. In addition, it can be seen that administration of GM1 (mTBI + GM1) prevented the reduction in memory performance received after injury (0.3 ± 0.04). Fisher LSD post-hoc analysis revealed ***p < 0.001. Statistical significance was examined by one-way ANOVA test [F(3,34) = 10.361; ***p < 0.001] followed by Fisher LSD post hoc.

As was shown before this damage to visual memory was found even 30 days post mTBI (−0.1 ± 0.08) compared with the control group (0.4 ± 0.06). Fisher LSD post-hoc analysis revealed ***p < 0.001. A single dose of GM1 (mTBI + GM1) prevented the reduction in memory following the injury (0.36 ± 0.06). Fisher LSD post-hoc analysis revealed ***p < 0.001. Statistical significance was tested by one-way ANOVA test [F (3,27) = 17.476; ***p < 0.001], followed by the LSD post hoc test.

#### Y-maze Test

According to the results of this test shown in Fig. 6, a significant deficit was observed in spatial memory in mTBI mice one week after injury (0.11 ± 0.07) in comparison to control mice (0.41 ± 0.06). Fisher LSD post-hoc analysis revealed *p < 0.05. The administration of GM1 (mTBI + GM1) prevented the cognitive decline measured one week after the injury (0.37 ± 0.08). Fisher LSD post-hoc analysis revealed *p < 0.05. Statistical significance was examined by one-way ANOVA test [F (3,43) = 4.897; *p < 0.05] followed by Fisher LSD post hoc. Next, we wanted to examine if GM1 prevents long-term cognitive damage as well. Using the same tests, additional mice were tested, one month after the injury.

Even 30 days after mTBI the damage in spatial memory was still significant (0.05 ± 0.05) compared to the control mice (0.35 ± 0.03). Fisher LSD post-hoc analysis revealed ***p < 0.001. GM1 administration (mTBI + GM1) prevented the cognitive decline measured one month after the injury (0.31 ± 0.03). Fisher LSD post-hoc analysis revealed; ***p < 0.001. Statistical significance was tested by one-way ANOVA test [F(3,33) = 7.670; ***p < 0.001. followed by Fisher LSD post hoc.

It is important to note that in both tests there was no significant difference in the GM1 group alone compared to the control group.

## Discussion

It was previously suggested that an alteration in the composition of gangliosides in the CNS, mainly the decrease in GM1 ganglioside, might lead to AOI (axonal outgrowth inhibition) by elevating the interaction of the myelin sheath with GD1a and GT1b on the axonal surface, and hence reduce the level of axonal regeneration^23–25^. These studies were done mainly on spinal cord injury. We have recently published that the same AOI mechanism was activated in the brains of a blast-TBI mouse model^18^. Moreover, even a single low dose of GM1 (2 mg/kg; IP) was sufficient to show neuroprotection following a blast-TBI in mice, both in regard to the axonal damage and to the cognitive impairments.

Following the above findings, the present work was aimed at finding whether this novel AOI mechanism due to the GM1 ganglioside reduction exists in the weight drop TBI model and whether this mechanism underlies the cellular, biochemical and cognitive deficits after traumatic brain injury.

The fact that the GM1 content in the brains of the mTBI injured mice was significantly reduced as seen by an indirect enzyme-linked immunoassay (ELISA) test and that GM1 injection just after the injury prevented this reduction (Fig. 2) supports our basic theory regarding the alteration in ganglioside composition post mTBI. This result is in agreement with our previous findings in blast-mTBI^18^. In addition, it has previously been suggested that reduction in GM1 content in the brain and a consequent decline in GM1/GD1 or GM1/GT1 ratios may lead to an elevated axon-myelin interaction and to AOI^13,17^. For this purpose it was important to examine the direct effect of the injury on regenerating neurons. We therefore measured the expression of the phosphorylated heavy neuro-filaments (pNF-H), which are found primarily in projections within the cortical and hippocampal axons. It has recently been suggested that these neurofilaments are involved in axonal regeneration^26–28^. Among animals, low pNF-H expression, neuronal injury, and other pathological conditions have been correlated with increased cell death^29^. It has also been previously reported that GM1 had a positive effect on neurofilaments expression^30,31^. Therefore, the expression of pNF-H can be a good measure of damage, as well as axonal recovery. Indeed, similarly to our previous report^18^ it can be seen that mTBI significantly reduced the expression of pNF-H in the cortex and hippocampus. Moreover, the administration of GM1 prevented this reduction (Fig. 3) which is in agreement with previous studies^30,31^.

Growth cones are dynamic structures supported by actin extensions which are found on the ends of growing axons and as such are used as axonal regeneration markers^32^. mTBI induced a significant decline in the appearance of growth cones in the cortices of the injured mice. This effect was prevented in GM1-treated mice, supporting the findings on the effect of mTBI on the pNF-H expression as was described above. These results are in agreement with our previous report regarding the effect of blast-TBI on the axonal regeneration^18^. In short, we can conclude that TBI inhibited the axonal regeneration in the injured brains, and that the fact that GM1 administration prevented this effect implies to the involvement of ganglioside alterations in this axonal outgrowth inhibition. Since the later experiments suggest that TBI induced a significant alteration in GM1ganglioside and that this effect underlies the AOI mechanism in the injured brains, it was essential to show that all of the described above led to neuronal death. Indeed, Fig. 5 shows that a significant reduction in neuronal survival was found in mTBI brains, as seen by NeuN staining. Similarly to the previous experiments, administration of GM1 prevented this reduction. These results reinforce our argument that mTBI inhibits axonal regeneration and even causes neuronal death, while GM1 administration immediately after the injury prevents these changes, indicating a link between the ganglioside levels and neuronal death. This effect also appears in the ischemic stroke model caused by obstruction of the Middle Cerebral Artery in rats. The results showed that the GM1 group significantly reduced autophagy activity which then resulted in increased neuronal survival^33^.

We have shown here images from the hippocampus and cortex. The cortical region that was being observed is the enthorinal cortex, EC (in the present as well as in previous studies). This region is the main interface between the hippocampus and neocortex. The EC-hippocampus system plays an important role in declarative (autobiographical/episodic/semantic) memories and in particular in spatial memory^34,35^. Thus, the cognitive tests presented in the following paragraph (Y-maze and NOR) can represent the function of these areas.

TBI-induced cognitive deficits are well known and studied in various animal models^36,37^. As expected, the injured mice in the present study performed poorly on the cognitive tests that included spatial and visual memories. Like our previous study regarding blast-TBI^18^, the administration of GM1 after the injury prevented these deficits. A single low dose of GM1 right after the injury not only prevented the cognitive damage 7 days post TBI but were successful in preventing damage also after 30 days. The protective effect of GM1 given close to the injury together with our findings regarding the decrease in GM1 content in the brain following the injury, may confirm our working hypothesis that a change in the ganglioside ratio (mainly the reduction in GM1 ganglioside) is part of the mechanism that underlies mTBI-induced damage, and that maintaining the levels of this ganglioside in the brain by its administration immediately after the injury prevents this pathway of AOI. GM1 was also found to be protective in another behavioral study where adult rats had bilateral lesion in the caudate nucleus^38^. In another model of brain injury by administering D-galactose it was found that GM1 injection significantly increased the proliferation, long-term survival and differentiation of hippocampal neurons. The location of the protective effect of GM1 implies to the correlation with improvement in the cognitive tests in mice^39^. In addition, in a mouse model with the degenerative and progressive Huntington’s disease (HD), *Intracerebroventricular* (ICV) *injection* of GM1 ganglioside resulted in inhibiting the neuronal toxic effects of the disease, and restoring normal motor functions to symptomatic mice^40^. Previous studies have shown that this injury does not alter motor abilities in our model: NSS (neurological severity score); The staircase was used to evaluate exploratory and locomotor activity; a novel grip strength procedure was developed for the purpose of evaluating neuromuscular fitness and fatigue; Further, a four-lane accelerating rotarod (San Diego Instruments, San Diego, CA) was used to evaluate motor learning^4,41^.

Further strengthening of the claim that GM1 is used as a neuroprotective factor was seen in a study that showed that after a head injury there was a decrease in the Mean Arterial Pressure (MAP) and an increase in the amount of water, Lactic Acid and Lipid Peroxidation. After GM1 injection, the MAP returned to normal values and the water content, LA and LPO decreased in the brain relative to the injured group^42^. More studies have shown the neuroprotective effect of GM1 regarding cholinergic axons, axonal survival and more, most of which were given in higher doses and for repetitive administration^43^.

Considering all the studies presented above, there is a strong basis to assume that the administration of GM1 ganglioside can inhibit the AOI mechanism following traumatic brain injury, thereby serving as a neuroprotective agent in various brain injuries. Since in the past decade or even earlier, TBI has been established as a major cause to CTE (chronic traumatic encephalopathy)^44–46^ new and novel potential treatments are needed to address this destructive process. Although GM1 have previously been used as a potential treatment for neurodegenerative diseases like PD and HD^40,47^, it is important to emphasize that in contrast to these reports where a high dose of GM1 or a longer range of treatment was needed to obtain desired results, this low-single dose was sufficient to prevent cognitive and biochemical damage. These results increase the range of treatment options after traumatic brain injury and demonstrate that early intervention after a head injury can prevent neurological deficits.
